# TIPE1‐mediated autophagy suppression promotes nasopharyngeal carcinoma cell proliferation via the AMPK/mTOR signalling pathway

**DOI:** 10.1111/jcmm.15550

**Published:** 2020-06-25

**Authors:** Yongliang Liu, Xiangqin Qi, Zhenan Zhao, Daoliang Song, Lianqing Wang, Qiaoli Zhai, Xiaoning Zhang, Peiqing Zhao, Xinxin Xiang

**Affiliations:** ^1^ Department of Otolaryngolgogy Zibo Central Hospital Shandong University Zibo China; ^2^ Department of Ultrasound Zibo Central Hospital Shandong University Zibo China; ^3^ Central of Translation Medicine Zibo Central Hospital Shandong University Zibo China

**Keywords:** AMPK/mTOR signalling pathway, autophagy, cell proliferation, nasopharyngeal carcinoma, TIPE1

## Abstract

Recent studies have shown that tumour necrosis factor‐α–induced protein 8 like‐1(TIPE1) plays distinct roles in different cancers. TIPE1 inhibits tumour proliferation and metastasis in a variety of tumours but acts as an oncogene in cervical cancer. The role of TIPE1 in nasopharyngeal carcinoma (NPC) remains unknown. Interestingly, TIPE1 expression was remarkably increased in NPC tissue samples compared to adjacent normal nasopharyngeal epithelial tissue samples in our study. TIPE1 expression was positively correlated with that of the proliferation marker Ki67 and negatively correlated with patient lifespan. In vitro, TIPE1 inhibited autophagy and induced cell proliferation in TIPE1‐overexpressing CNE‐1 and CNE‐2Z cells. In addition, knocking down TIPE1 expression promoted autophagy and decreased proliferation, whereas overexpressing TIPE1 increased the levels of pmTOR, pS6 and P62 and decreased the level of pAMPK and the LC3B. Furthermore, the decrease in autophagy was remarkably rescued in TIPE1‐overexpressing CNE‐1 and CNE‐2Z cells treated with the AMPK activator AICAR. In addition, TIPE1 promoted tumour growth in BALB/c nude mice. Taken together, results indicate that TIPE1 promotes NPC progression by inhibiting autophagy and inducing cell proliferation via the AMPK/mTOR signalling pathway. Thus, TIPE1 could potentially be used as a valuable diagnostic and prognostic biomarker for NPC.

## 1 INTRODUCTION

Nasopharyngeal carcinoma (NPC), which is derived from nasopharyngeal epithelial cells, is the most prevalent head and neck cancer in certain regions of East Asia and southern China.^1^ NPC presents with the potential for local invasion and early lymph node or distant organ metastasis.^2^ Due to the specific anatomical location and relatively high sensitivity to radiation of NPC, many NPC patients are diagnosed at the late stage, and radiotherapy remains the primary treatment.^3^ Despite modern diagnostic imaging and advanced radiotherapy technology, 20%‐30% of NPC patients still develop locoregional recurrence or distant organ metastasis.^4^ Therefore, the identification of novel molecular mechanisms underlying NPC pathogenesis is urgently needed to further predict tumour progression and prognosis in NPC patients.

Tumour necrosis factor‐α–induced protein 8 like‐1 (TIPE1), also known as Oxi‐β, belongs to the tumour necrosis factor‐α–induced protein 8 (TNFAIP8) family. TIPE1 was identified in 2008 and plays crucial roles in modulating immunity and tumorigenesis.^5^, ^6^, ^7^ TIPE1 negatively regulates dendritic cell maturation and T cell immunity by inhibiting the programmed death ligand 1/programmed cell death protein 1 signalling pathway in sepsis.^8^ Previous studies showed that TIPE1 could induce apoptosis in RAW264.7 cells or hepatocellular carcinoma cells by increasing the expression of Bcl‐2 family proteins or down‐regulating the Rac1 pathway,^9^, ^10^ and TIPE1 may also be a novel target capable of modulating tumorigenesis in gastric and lung cancer.^11^, ^12^ Furthermore, the results of our previous study indicated that TIPE1 restricted p53 acetylation to play an oncogenic role in cervical cancer.^13^ However, the biological function of TIPE1 in NPC has not been fully evaluated.

Autophagy is a highly conserved and complex self‐digestion process that maintains cellular homeostasis, plays an important role in cell survival and is intimately related to tumorigenesis.^14^, ^15^, ^16^ A previous study demonstrated that autophagy was also involved in NPC. An increased level of hypoxia‐inducible factor‐1α–associated beclin‐1 protein was shown to be associated with poor overall survival in NPC, suggesting that this protein may be a novel prognostic biomarker for overall survival and a therapeutic molecular target.^17^ Zhu *et al* showed that annexin A1 inhibited autophagy to promote NPC metastasis via the phosphoinositide 3‐kinase/Akt signalling pathway.^18^ In another study, the down‐regulation of cyclinB1 expression induced autophagy by increasing reactive oxygen species levels via the activated AMP‐activated protein kinase(AMPK)‐Unc‐51‐like kinase 1 (ULK1)‐dependent signalling pathway in human NPC cell lines.^19^ However, few studies have explored the relationship between TIPE1 and autophagy, with only one such study having been reported to date, the results of which showed that oxidative stress‐induced TIPE1 stabilized the tuberous sclerosis complex 2 (TSC2) protein to promote autophagy in Parkinson's disease.^20^ To explore the role of TIPE1 in NPC, in this study, we investigated whether TIPE1 affects the biological behaviour of NPC via the autophagy pathway and assessed the potential molecular mechanism.

## 2 MATERIALS AND METHODS

### 2.1 Study group

One hundred eight cases of NPC biopsy tissue samples and normal nasopharyngeal epithelium tissue samples collected between 2004 and 2018 were selected from the pathology database of Zibo Central Hospital. Two microarray blocks, including 68 NPC tissue samples and 40 chronic rhinosiusitis tissue samples, were constructed by re‐embedding paraffin‐embedded tissue samples. This research was approved by the Shandong University Medical Ethics Committee according to the Declaration of Helsinki, and all patients provided informed consent.

### 2.2 Cell culture and the construct ion of stable TIPE1‐overexpressing or TIPE1‐knockdown NPC cell lines

The cell lines CNE‐1 and CNE‐2Z were purchased from the Shanghai Institute of Cell Biology and Cell Bank (Chinese Academy of Sciences Committee, Shanghai, China) and cultured in RPMI 1640 medium (Gibco, Grand Island, USA) supplemented with 10% foetal bovine serum (Gibco, Grand Island, USA) under a 5% CO_2_ humidified atmosphere at 37°C.

Lentiviral vectors expressing TIPE1 (Lv‐TIPE1), TIPE1 shRNA (Sh‐TIPE1) or a scrambled non‐targeted shRNA (Sh‐Scr) were constructed and confirmed through sequencing by GeneChem Company (Shanghai, China). CNE‐1 or CNE‐2Z cells were infected with Lv‐TIPE1, Sh‐TIPE1 and their control lentiviral vectors and selected with puromycin for 2 weeks to establish stable TIPE1‐overexpressing or TIPE1‐knockdown NPC cell lines.

### 2.3 RFP‐LC3 stable cell lines and quantitative RFP‐LC3 analyses

A stable CNE‐1 RFP‐LC3 stable cell line was established by transient transfection of the Ubi‐mTagRFP‐LC3 lentiviral vector (GeneChem Company, Shanghai, China). RFP‐LC3 puncta formation was determined by capturing images using a Leica ICC50 HD microscope(Leica Microsystems, Wetzlar, Germany). Cells were fixed and stained with DAPI for nuclear visualization. To quantify autophagic cells, we counted the number of autophagic cells in 100 fields as determined by observing RFP‐LC3 puncta (20 puncta indicated a positive cell).

### 2.4 Cell proliferation and cell cycle analysis

Cell Counting Kit‐8 (CCK8, Dojindo, Shanghai, China) assays were performed to detect the proliferation and viability of CNE‐1 and CNE‐2Z cells on different days according to the manufacturer's instructions. For colony formation assays, CNE‐1 or CNE‐2Z cells (1 × 10^3^ cells per well) were seeded in 6‐well plates and cultured for 7 days. The number of colonies was counted, and the plates were imaged after most of the colonies contained more than 50 cells. To analyze the cell cycle, CNE‐1 and CNE‐2Z cells were harvested, fixed and stained with propidium iodide (PI; Sigma, USA) for 30 minutes in the dark. The cells (10 000 events) were evaluated with a flow cytometer (FACS Aria 2, BD Biosciences, USA).

### 2.5 Microarray analysis

CNE‐2Z cells infected with Lv‐TIPE1 or the control vector were harvested to analyze gene expression using an Affymetrix GeneChip® 2.0 ST array. According to the manufacturer's instructions, experiments were performed by the GeneChem Company. Kyoto Encyclopedia of Genes and Genomes (KEGG) pathway mapping and Gene Ontology (GO) analysis were used to analyze the data sets.

### 2.6 Transmission electron microscopy (TEM)

CNE‐1 and CNE‐2Z cells infected with Lv‐TIPE1 or the control vector were fixed with 3% glutaraldehyde for 2 hours, incubated with 1% osmium in a 0.1% cacodylate buffer at 4°C for 1.5 hours, dehydrated in a series of graded acetone solutions and then embedded in LX‐112 medium (Ladd Research Industries, Inc). After polymerization, ultrathin sections were cut with an MT‐7000 ultramicrotome (RMC Products, Tucson, USA). Subsequently, the sections were stained with 4% uranyl acetate and lead citrate, and digital images were obtained with a JEM1230 electron microscope (Horiba Corp, Kyoto, Japan).

### 2.7 Western blotting

Protein extracts from cultured cells or tumour tissue samples were immunoblotted with antibodies against TIPE1 (sc‐82761, Santa Cruz, USA), Ki67 (ab15580, Abcam, USA), phosphorylated (p‐) mTOR (5536, Cell Signaling Technology (CST), Beverly, USA), mTOR (2983, CST, Beverly, USA), pS6 (4858, CST, Beverly, USA), S6 (2217, CST, Beverly, USA), TSC2 (4305, CST, Beverly, USA), LC3B (3868, CST, Beverly, USA), P62 (39 749, CST, Beverly, USA) and GAPDH (sc‐25778, Santa Cruz, USA) as previously described.^21^ After incubating with HRP‐conjugated secondary antibodies, the specific reaction was visualized using a Tanon imaging system (Tanon Company, Shanghai, China). Quantification of image densities was analysed using ImageJ (NIH, Bethesda, USA).

### 2.8 Immunohistochemical staining

Immunohistochemical staining for p‐S6, LC3B, TIPE1 and Ki67 was performed as previously described.^21^ The staining was assessed separately via a German semiquantitative scoring system to evaluate target protein expression. Six fields of view per slide were independently counted by two pathologists. Control IgG was used as a negative control instead of the specific primary antibody under the same conditions.

The immunohistochemical staining scores were assigned a mean score based on both the intensity of staining and the proportion of tumour cells with an unequivocal positive reaction. Each section was independently assessed by two pathologists lacking prior knowledge of patient data. Positive reactions were defined as those showing brown signals in the cell cytoplasm. Staining index values (0‐12) were determined by multiplying the score for staining intensity with the score for positive area. The intensity was scored as follows: 0 = negative; 1 = weak; 2 = moderate; and 3 = strong. The frequency of positive cells was defined as follows: 0 = less than 5% stained; 1 = 5 to 25% stained; 2 = 26 to 50% stained; 3 = 51 to 75% stained; and 4 = greater than 76% stained. The Ki‐67 index was assessed by the proportion of tumour cells with an unequivocal positive reaction.

### 2.9 In vivo xenograft mouse model

BALB/c nude mice (male, 4‐6 weeks old) were purchased from the Shanghai Laboratory Animal Co.(SLAC; Shanghai, China) and housed under specific pathogen‐free conditions with the approval of the Animal Care and Use Committee of Shandong University. For tumorigenesis assays, 4 × 10^6^ CNE‐2Z cells in PBS (Lv‐TIPE1‐ or control vector‐infected) were injected subcutaneously into the right posterior limb of nude mice. When the tumours reached an average volume of approximately 80 mm^3^, tumour volumes were measured with calipers every three days. After 16 days, the mice were killed, and the tumours were collected, weighed and imaged.

### 2.10 Statistical analysis

The data are presented as the means ± SEM and were analysed with GraphPad Prism (GraphPad Software, San Diego, USA). Two‐way ANOVA, Student's t test, Kaplan‐Meier survival analysis or log‐rank test was used as appropriate. A *p* value < 0.05 was considered to be significant.

## 3 RESULTS

### 3.1 Elevated TIPE1 expression is associated with a poor prognosis in patients with NPC

**FIGURE 1:**
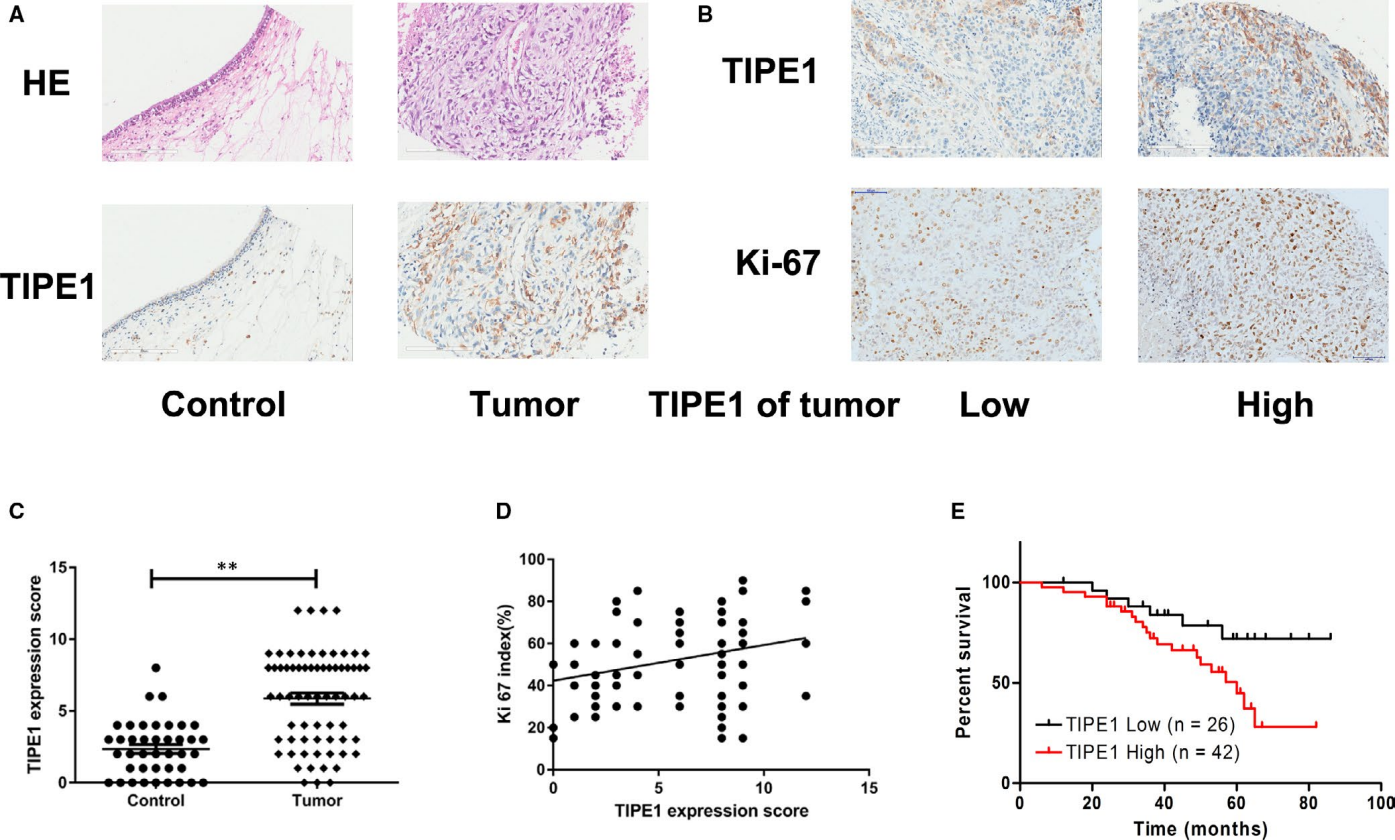
Elevated TIPE1 expression in nasopharyngeal carcinoma tissue samples is related to a poor prognosis in NPC patients. A and C, Representative immunohistochemical staining images and the staining intensity of TIPE1 expression in nasopharyngeal carcinoma and control tissue samples. B and D, Protein expression of TIPE1 and Ki67 in tumours and the associated relationship. E, Overall survival rates of NPC patients with high or low TIPE1 expression. Scale bars: 200 μm.**P < .01

To confirm the role of TIPE1 in NPC, we first investigated the expression of TIPE1 in NPC and control tissue samples by immunohistochemical staining. As shown in Figure 1A and C, TIPE1 levels were dramatically higher in the NPC tissue samples than in the normal nasopharyngeal epithelial tissue samples (*P* < .001) (Figure 1A and C). Moreover, the expression of TIPE1 was positively correlated with that of the proliferation marker Ki67 (*r* = 0.2835, *P* = .019) (Figure 1B and D), and the expression of TIPE1 in the NPC tissue samples was negatively correlated with the overall survival rate of the patients (*P* = .039) (Figure 1E). NPC patients with high TIPE1 levels had higher Ki67 expression and a shorter lifespan than those with low TIPE1 expression, indicating that elevated TIPE1 levels predict the poor prognosis.

### 3.2 TIPE1 promotes NPC cell proliferation in vitro

**FIGURE 2:**
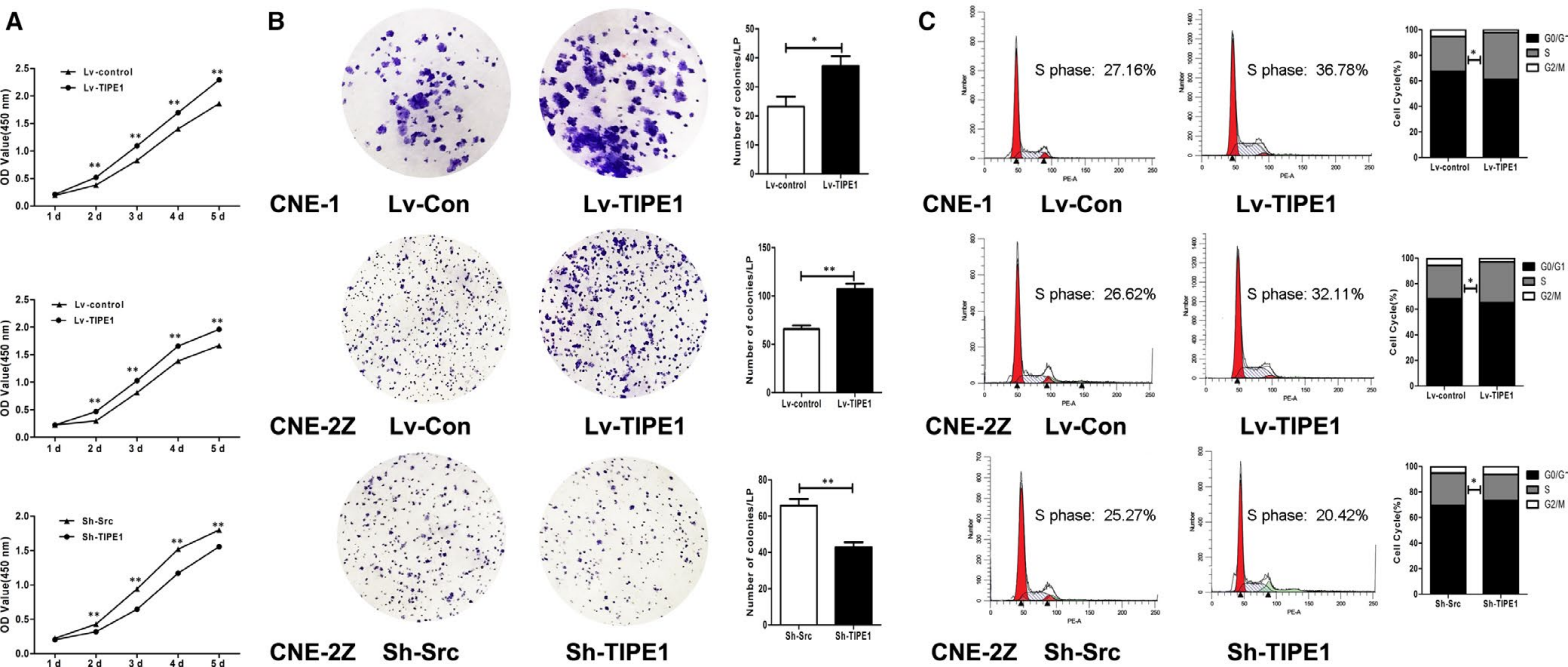
TIPE1 promotes nasopharyngeal carcinoma cell proliferation in vitro. The human NPC cell lines CNE‐1 and CNE‐2Z were infected with Lv‐TIPE1 and Lv‐control or Sh‐TIPE1 and Sh‐Scr. A, The CCK8 assay was used to assess cell growth. B, Representative images show colony formation, and the colonies were counted. C, Representative images show the cell cycle progression, as assessed by flow cytometry. The data are presented as the means ± SD, *P < .05, **P < .01; the experiments were repeated at least three times

To further investigate the biological role of altered TIPE1 expression in NPC cells, CCK8 assays, colony formation assays and flow cytometry were performed. A lentiviral vector expressing TIPE1 (Lv‐TIPE1) or a control vector (Lv‐control) was used to infect CNE‐1 and CNE‐2Z cells. The CCK8 and colony formation assay results showed that the cell proliferation and number of colonies of CNE‐1 and CNE‐2Z cells increased after TIPE1 was overexpressed (*P* < .05) (Figure 2A and B). Furthermore, the percentage of cells in S phase detected by flow cytometry was increased after TIPE1 was overexpressed (*P* < .05) (Figure 2C). In addition, knocking down TIPE1 expression with Sh‐TIPE1 infection significantly decreased cell proliferation and decelerated cell cycle progression in CNE‐2Z cells (Figure 2A, B and C). These findings indicate that TIPE1 promotes proliferation and growth in CNE‐1 and CNE‐2Z cells.

### 3.3 TIPE1 inhibits autophagy in NPC cells

**FIGURE 3:**
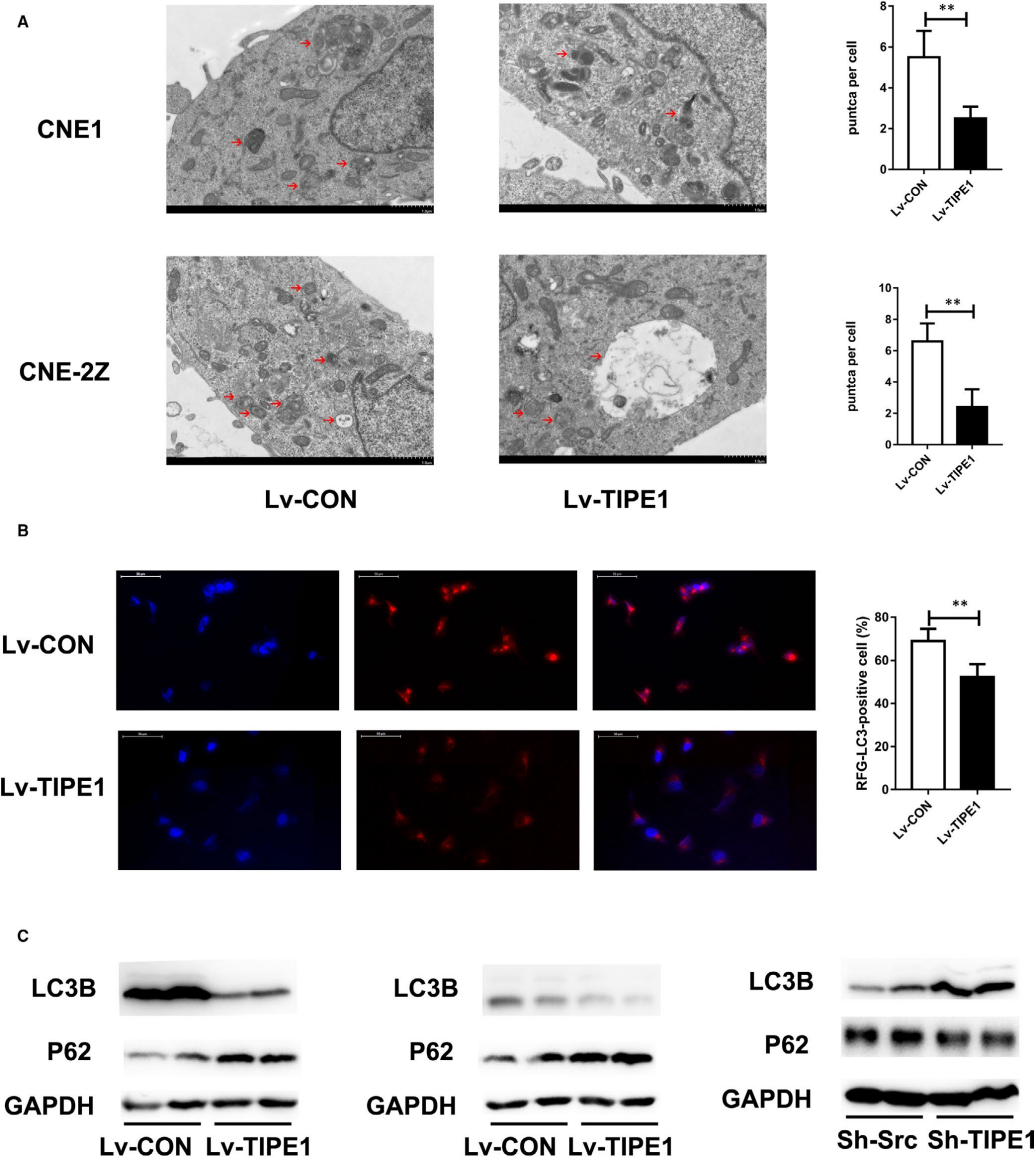
TIPE1 inhibits autophagy in NPC cells. A, TEM results showing the autophagosome density in TIPE1‐overexpressing or control CNE‐1 and CNE‐2Z cells. Representative images are shown. Scale bar: 1 μm. Quantitative analysis of puncta is shown in the right panel. B, Ubi‐mTagRFP‐LC3 infection resulted in LC3 puncta in CNE‐1 cells. Representative images are shown. Scale bar: 50 μm. A quantitative analysis of RFP‐LC3B‐positive cells is shown in the right panel. C, Representative Western blotting results showing that the expression of autophagy‐associated proteins in CNE‐1 cells after TIPE1 was overexpressed. D, Representative Western blotting results showing the expression of autophagy‐associated proteins in CNE‐2Z cells after TIPE1 was overexpressed. E, Representative Western blotting results showing the expression of autophagy‐associated proteins in Sh‐TIPE1–infected or Sh‐Scr–infected CNE‐2Z cells. Scale bars: 1 μm. The data are presented as the means ± SD, *P < .05, **P < .01; the experiments were repeated at least three times

TEM analysis showed that autophagosome density was significantly decreased in TIPE1‐overexpressing CNE‐1 and CNE‐2Z cells (*P* < .01) (Figure 3A). To further confirm that TIPE1 inhibits autophagy in NPC cells, we investigated the inhibition of autophagy by RFP‐LC3 transient transfection to localize the autophagosome‐specific protein LC3. As shown in Figure 3B, we observed that the number of autophagosomes in TIPE1‐overexpressing CNE‐1 cells was reduced. Overexpressing TIPE1 significantly decreased the protein level of LC3B and promoted that of P62 in CNE‐1 and CNE‐2Z cells (Figure 3C and D). Furthermore, Sh‐TIPE1 increased the protein level of LC3B and reduced that of P62 (Figure 3E). These results demonstrate that TIPE1 inhibits autophagy in CNE‐1 and CNE‐2Z cells.

### 3.4 TIPE1 inhibits autophagy in NPC cells through the AMPK/mTOR signalling pathway

**FIGURE 4:**
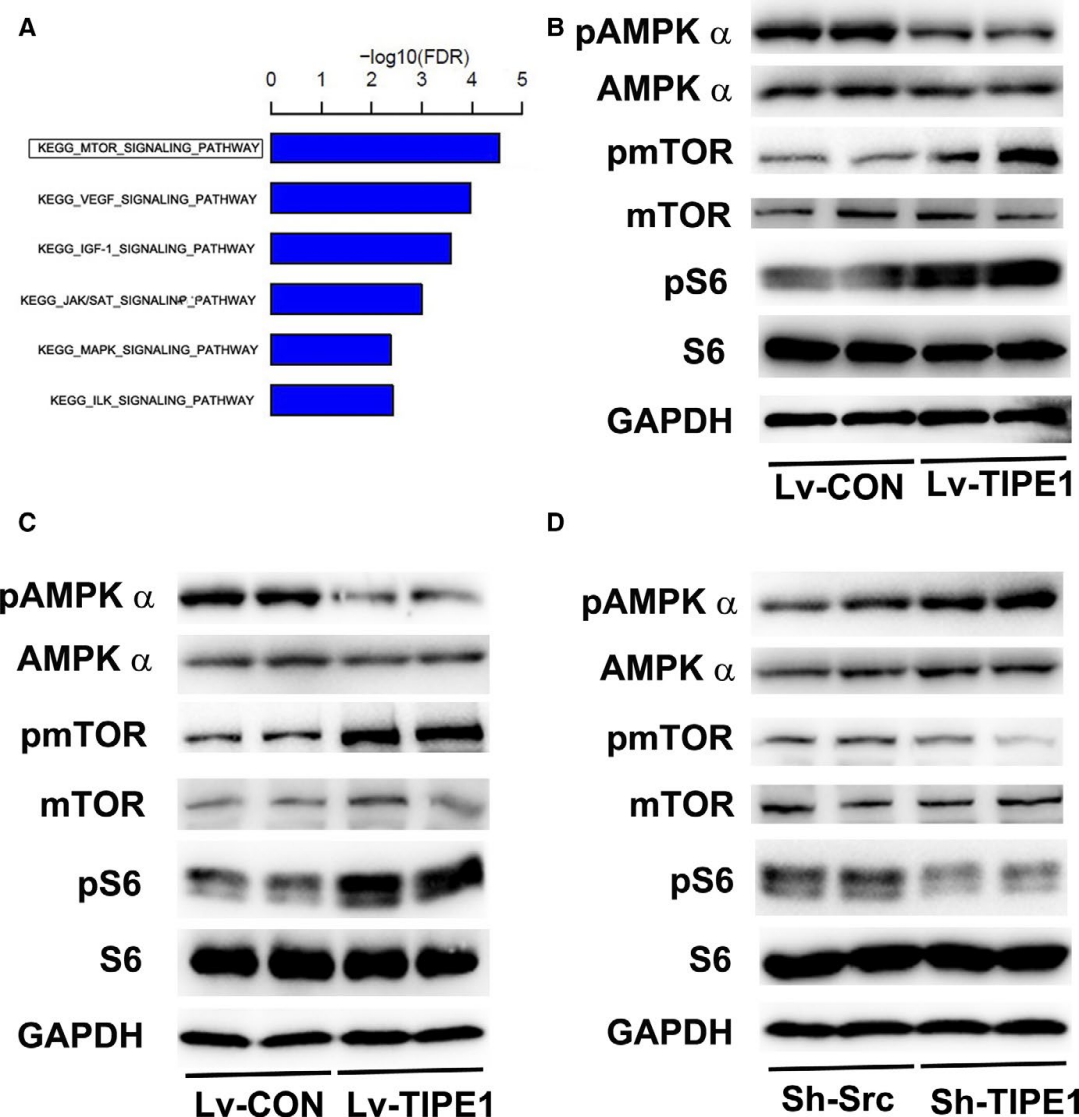
TIPE1 modulates the AMPK/mTOR signalling pathway. A, KEGG pathway mapping was used to perform pathway analysis of CNE‐2Z cells infected with Lv‐TIPE1 or Lv‐control. B, Representative Western blotting results showing the expression of proteins in the AMPK/mTOR signalling pathway in CNE‐1 cells after TIPE1 was overexpressed. C, Representative Western blotting results showing the expression of proteins in the AMPK/mTOR signalling pathway in CNE‐2Z cells after TIPE1 was overexpressed. D, Representative Western blotting results showing the expression of proteins in the AMPK/mTOR signalling pathway in Sh‐TIPE1–infected or Sh‐Scr–infected CNE‐2Z cells. The data are presented as the means ± SD, *P < .05, **P < .01; the experiments were repeated at least three times

To further elucidate the signalling pathway involved in TIPE1‐associated autophagy in NPC, Affymetrix GeneChip® arrays were performed for CNE‐2Z cells infected with Lv‐TIPE1 or Lv‐control (GEO accession number is GSE 147 252). The data demonstrated that the downstream mTOR signalling pathway was up‐regulated in the Lv‐TIPE1 group compared to that observed in the Lv‐control group (Figure 4A). To further confirm this finding, we investigated the protein expression of candidate components in CNE‐1 and CNE‐2Z cells infected with Lv‐TIPE1 or Sh‐TIPE1. TIPE1 overexpression dramatically increased the protein expression of molecules in the mTOR signalling pathway and decreased that of AMPKα, a negative regulator of mTOR (Figure 4B and C). Furthermore, Sh‐TIPE1 down‐regulated the protein expression of molecules in the mTOR signalling pathway and increased that of AMPKα (Figure 4D). These findings show that the AMPK/mTOR signalling pathway is involved in TIPE1‐associated autophagy inhibition in NPC.

**FIGURE 5:**
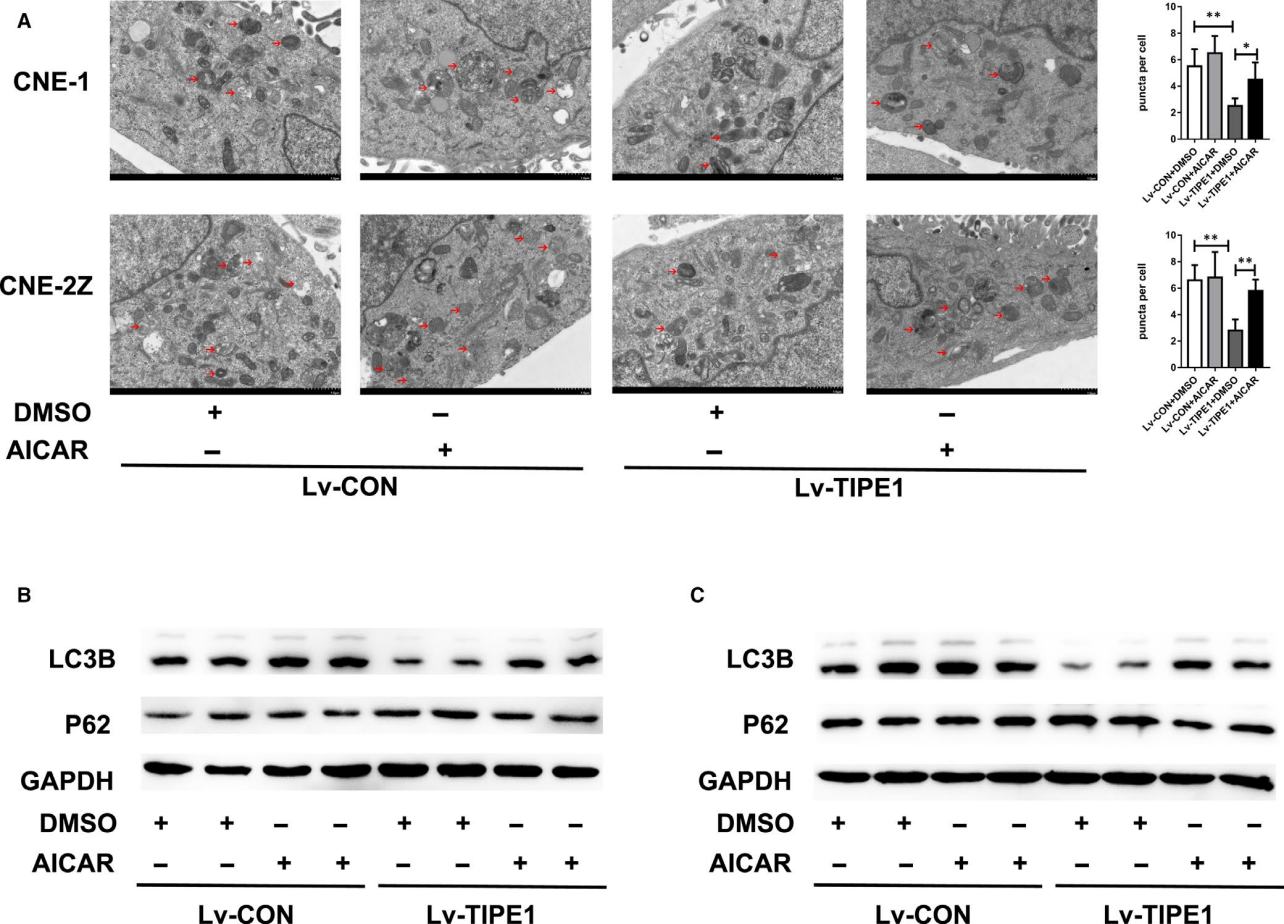
AICAR reverses the TIPE1‐mediated decrease in autophagy. A, TEM analysis showing the autophagosome density in TIPE1‐overexpressing CNE‐1 and CNE‐2Z cells treated with AICAR (500 μM) or a vehicle for 24 hr B, Western blotting results showing the levels of autophagy‐associated proteins in TIPE1‐overexpressing CNE‐1 treated with AICAR (500 μM) or a vehicle for 24 hr C, Western blotting results showing the levels of autophagy‐associated proteins in TIPE1‐overexpressing CNE‐2Z cells treated with AICAR (500 μM) or a vehicle for 24 hr Scale bars: 1 μm. The data are presented as the means ± SD, *P < .05, ** *P *<.01; the experiments were repeated at least three times

To further investigate the role of the AMPK/mTOR signalling pathway in cell autophagy, TIPE1‐overexpressing CNE‐1 and CNE‐2Z cells were treated with the AMPK activator 5‐amino‐1‐β‐D‐ribofuranosyl‐1H‐imidazole‐4‐carboxamide (AICAR; HY‐13417, MCE, New Jersey, USA). TEM results showed that compared with the control treatment, the AICAR treatment significantly increased the autophagosome density in TIPE1‐overexpressing CNE‐1 and CNE‐2Z cells (Figure 5A). Furthermore, the protein level of LC3B was significantly increased, whereas that of P62 was decreased after the AICAR treatment (Figure 5B and C). These results indicate that AICAR can reverse the TIPE1‐mediated decrease in autophagy. In summary, TIPE1 regulates the AMPK/mTOR signalling pathway to modulate autophagy in NPC.

### 3.5 TIPE1 promotes tumour growth by inhibiting autophagy via the AMPK/mTOR signalling pathway in BALB/c nude mice

**FIGURE 6:**
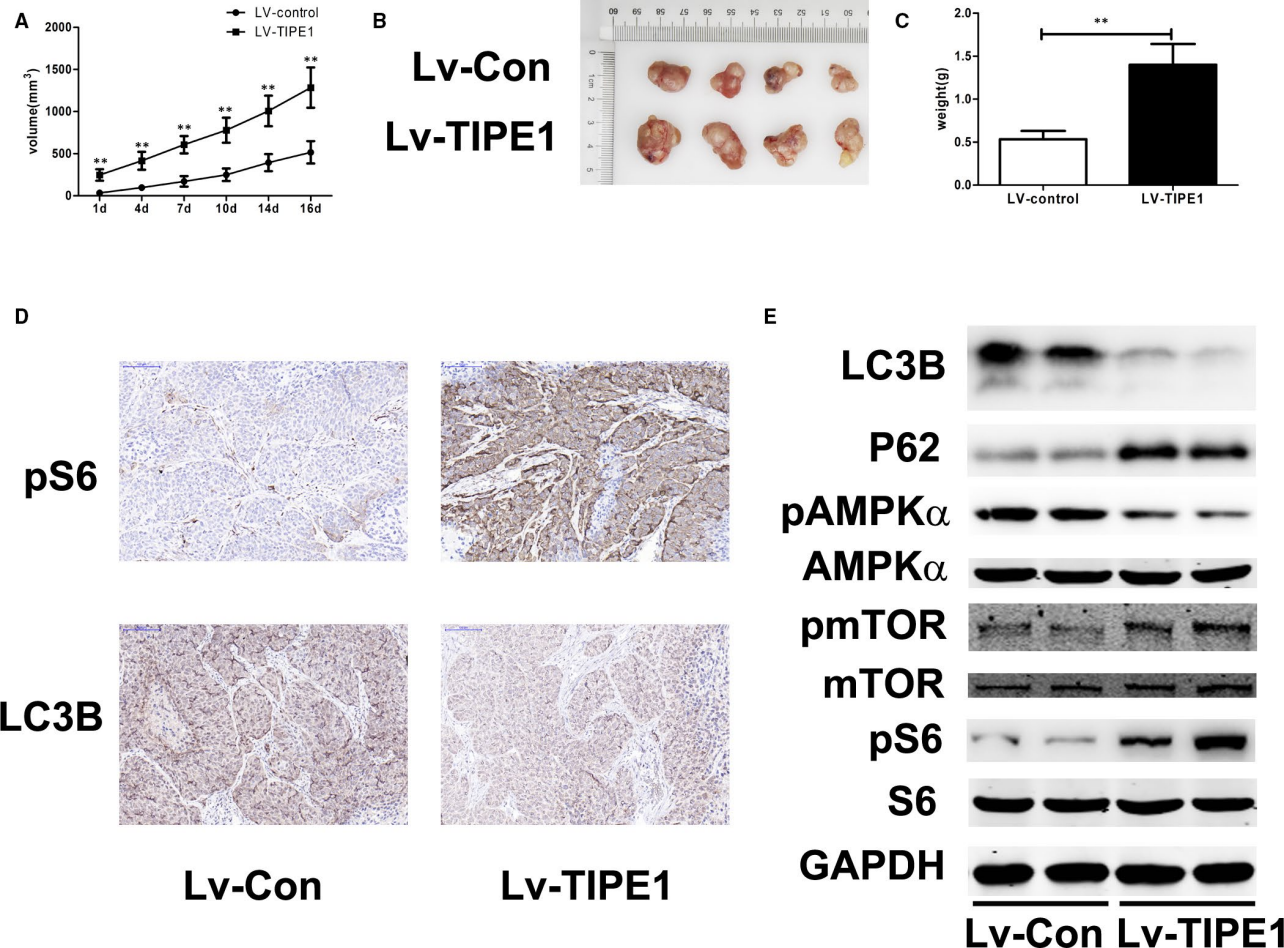
TIPE1 induces tumorigenesis and inhibits autophagy in NPC cells in vivo. CNE‐2Z cells stably infected with Lv‐TIPE1 or Lv‐control were injected subcutaneously into nude mice. A, Changes in tumour volume, B, tumour images and C, tumour weights are shown. D, Representative immunohistochemical images of pS6 and LC3B protein levels in xenograft tumour tissue samples are shown. E, Representative Western blotting results showing the protein expression of an autophagy‐associated protein and proteins in the AMPK/mTOR signalling pathway. Scale bars: 100 μm. The data are presented as the means ± SD, *P < .05, **P < .01 (n = 6)

To further explore the biological role of TIPE1 in NPC in vivo, a tumorigenicity assay was performed in nude mice by subcutaneously transplanting CNE‐2Z cells infected with Lv‐TIPE1. The resulting tumour growth curve demonstrates that tumour growth in the Lv‐TIPE1 group was significantly promoted compared to that observed in the control group (Figure 6A and B). In addition, the tumour weights in the Lv‐TIPE1 group were also substantially increased at the time of sacrifice compared to those observed in the Lv‐control group (*P* < .01) (Figure 6C). Immunohistochemical analysis showed that the expression of LC3B in tumour tissue was reduced in the Lv‐TIPE1 group compared to that observed in the Lv‐control group, whereas the expression of pS6 was increased (Figure 6D). In addition, the protein expression of LC3B was decreased and that of P62 was increased in the Lv‐TIPE1 group, indicating that TIPE1 inhibited autophagy (Figure 6E). Compared to that observed in the Lv‐control group, the expression of pmTOR and pS6 expression was highly up‐regulated in the Lv‐TIPE1 group, while pAMPKα expression was down‐regulated in the Lv‐TIPE1 group (Figure 6E). These results indicate that TIPE1 inhibits autophagy via AMPK/mTOR in vivo.

## 4 DISCUSSION

The results of this show that TIPE1 promotes NPC progression by inducing cell proliferation and inhibiting autophagy via the AMPK/mTOR signalling pathway. Our conclusion is supported by the following observations: (a) TIPE1 expression was remarkably up‐regulated in NPC tissue samples compared with normal nasopharyngeal epithelial tissue samples; (b) the expression of TIPE1 was positively correlated with that of the proliferation marker Ki67 and negatively correlated with the lifespan of NPC patients; (c) in vitro, TIPE1 induced cell proliferation and inhibited autophagy in TIPE1‐overexpressing CNE‐1 and CNE‐2Z cells; (d) knocking down TIPE1 expression induced autophagy and decreased proliferation; (e) overexpressing TIPE1 increased the protein expression of pmTOR, pS6 and P62 and decreased that of pAMPKα and LC3B; (f) the decrease in autophagy was remarkably rescued in TIPE1‐overexpressing CNE‐1 and CNE‐2Z cells treated with the AMPK activator AICAR; and (g) TIPE1 promoted tumour growth in nude mice.

Previous studies have shown that TIPE1 is expressed in hepatocytes, muscle tissues, neurons, germ cells and a variety of epithelial cells.^22^ TIPE1 is also present in many cancer cells of epithelial origin, including breast, cervical, bladder and gastric cancer cells.^11^, ^13^, ^22^, ^23^, ^24^, ^25^ To date, a great deal of evidence has shown that TIPE1 plays crucial roles in the carcinogenesis of many cancers, including hepatocellular carcinoma, gastric cancer, lung cancer, osteosarcoma, cervical cancer, colon cancer and breast cancer. TIPE1 can induce apoptosis in RAW264.7 and hepatocellular carcinoma cells by increasing the levels of Bcl‐2 family proteins or down‐regulating the Rac1 pathway.^10^ Furthermore, TIPE1 can serve as a potential molecular target in breast, gastric and lung cancer, as it is capable of modulating tumour growth and metastasis. TIPE1 can also impair the stemness of colorectal cancer by directly targeting β‐catenin.^26^ Indeed, TIPE1 induces cell apoptosis and inhibits cell proliferation and tumorigenesis. In contrast, TIPE1 restricts p53 acetylation to play an oncogenic role in cervical cancer. In summary, the biological function of TIPE1 is controversial, and its mechanism of action remains to be fully elucidated. For the first time, we examined the expression of TIPE1 in nasopharyngeal epithelial cells in clinical tissue samples. Our study showed that TIPE1 expression was remarkably promoted in NPC tissue samples compared to control nasopharyngeal epithelial tissue samples. Moreover, the expression of TIPE1 was positively correlated with that of the proliferation marker Ki67 and negatively correlated with the lifespan of NPC patients. In vitro, TIPE1 promoted cell proliferation in TIPE1‐overexpressing CNE‐1 and CNE‐2Z cells. Concordantly, knocking down TIPE1 expression decreased cell proliferation. Consistent with the results of our previous research in cervical cancer, TIPE1 may serve as an oncogene in NPC.

Autophagy has been shown to be intimately related with cancer.^27^, ^28^, ^29^ In this study, the decreased density of characteristic autophagosomes and the decreased expression of LC3B in tumour tissue sections provided strong evidence that autophagy was inhibited after TIPE1 was overexpressed. The AMPK/mTOR signalling pathway is a primary and key pathway in autophagy regulation that can coordinately determine the survival and autophagy of cancer cells and play a vital role in tumorigenesis.^30^, ^31^, ^32^ To characterize the unique functions of TIPE1 in autophagy in NPC, we performed GeneChip arrays to analyze the related signalling pathway after overexpressing TIPE1 in CNE‐2Z cells. The data indicated that the AMPK/mTOR signalling pathway was remarkably increased. Further results demonstrated that TIPE1 overexpression dramatically increased the protein expression of molecules in the mTOR signalling pathway and decreased that of AMPKα, a negative regulator of mTOR. Ji‐Young Ha *et al* showed that 6‐hydroxydopamine‐induced oxidative stress increased the expression of TIPE1, leading to cellular autophagy and death in neuronal cell lines by stabilizing TSC2^20^. The results of our study showed that there was no change in the mRNA and protein levels of TSC2 in CNE‐1 and CNE‐2Z cells after TIPE1 overexpression (data not shown). Furthermore, the decrease in autophagy was remarkably rescued in TIPE1‐overexpressing CNE‐1 and CNE‐2Z cells treated with the AMPK activator AICAR. These data show that TIPE1 regulates the AMPK/mTOR signalling pathway to modulate autophagy in NPC.

In summary, the results of our study indicate that TIPE1 inhibits autophagy via the AMPK/mTOR signalling pathway in NPC. For the first time, we observed that TIPE1 was dramatically associated with overall lifespan in NPC patients and promoted NPC cell proliferation in vivo and in vitro. Thus, TIPE1 may be a novel and valuable biomarker for NPC diagnosis and prognosis.

## CONFLICT OF INTEREST

There are no conflicts of interest to declare.

## AUTHOR CONTRIBUTIONS

YL Liu, XQ Qi, ZA Zhao, DL Song, LQ Wang, XN Zhang and QL Zhai performed the experiments; YL Liu, PQ Zhao and XX Xiang analysed the data; PQ Zhao and XX Xiang designed the experiments and wrote the paper.

## Data Availability

The data that support the findings of our study are available from the corresponding author upon reasonable request.
